# Reply to Muntean et al. Comment on “Mitteregger et al. A Variant Carbapenem Inactivation Method (CIM) for *Acinetobacter baumannii* Group with Shortened Time-to-Result: rCIM-A. *Pathogens* 2022, *11*, 482”

**DOI:** 10.3390/pathogens11070767

**Published:** 2022-07-05

**Authors:** Dieter Mitteregger, Julian Wessely, Ivan Barišić, Branka Bedenić, Dieter Kosak, Michael Kundi

**Affiliations:** 1 Department of Clinical Microbiology, Laboratory Dr. Kosak, Dr. Reckendorfer and Partner, 1090 Vienna, Austria; julian2012@hotmail.com; 2 Molecular Diagnostics, Center for Health & Bioressources, AIT Austrian Institute of Technology GmbH, 2444 Vienna, Austria; ivan.barisic@ait.ac.at; 3 School of Medicine, University of Zagreb, 10000 Zagreb, Croatia; bbedenic@mef.hr or; 4 Clinical Department of Clinical and Molecular Microbiology, University Hospital Center Zagreb, 10000 Zagreb, Croatia; 5 Department of Clinical Pathology, Laboratory Dr. Kosak, Dr. Reckendorfer and Partner, 1090 Vienna, Austria; dr.kosak@dr-kosak.at; 6 Center for Public Health, Department of Environmental Health, Medical University of Vienna, 1090 Vienna, Austria; michael.kundi@meduniwien.ac.at

We appreciate the interest in our publication and agree that a different acronym for the method would have been better. However, as stated in the title, the method is designed for the *Acinetobacter baumannii* group and not for *Enterobacterales,* for which a modification was described by Muntean, A.-A. et al. in August 2021: “Optimization of the rapid carbapenem inactivation method for use with AmpC hyperproducers” [1]. It has been well known, since the research conducted by Simner et al. (2018), that different approaches must be used for *A. baumannii* and *Enterobacterales* [2]. Thus, from our point of view, there is a very low risk of confusing the methods, despite them having the same acronym.

In their comment, Muntean, A.-A. et al. [3] describe the differences between the method of van der Zwaluw et al. [4] and our protocol [5], which are essentially the reasons why the sensitivity and specificity both resulted in only 83% for *A. baumannii* by the otherwise seminal approach of van der Zwaluw et al. Again, this confirms the requirement for variation in the methods used for these very different bacteria.

Similarly, the large investigation by the French National Reference Center dealt with *Enterobacterales*, not *Acinetobacter* spp. [6].

In our newly described “carryover-microsatellites” [5], the position information is of vital importance. Two out of three phenomena were previously described by Pierce et al. (2017) [7]. In Figure 2 of [7], pinpoint colonies of the read-out strain, statistically distributed throughout the zone of inhibition, are shown; whereas, in their Figure 3B, the narrow ring of growth abutting the disk is explained as carryover of the test organism. Scattered growth of the read-out strain, as depicted in their Figure 1, was indicative of positive results, depending on the width of the inhibition zone. Carryover growth of the test organism had no impact on test interpretation. The third phenomenon was described in our recent publication [5]. The differences between this phenomenon and the previously described phenomena are: the colonies do not represent the test strain and are not statistically distributed throughout the inhibition zone, but surround the carryover growth of the (carbapenemase-positive) test strain; hence, we described this as “carryover-microsatellites” (Figure 2, [5]). This finding revealed the presence of carbapenemase genes in a few cases, despite the inhibition zones being above the respective cut-off, and was reproducible in triplicate repetitions of the entire procedure.

Our discussion of the possibility of collective incubation of three carbapenem disks was based on chemical considerations of lactamase action. In the final catalytic step, a strategically positioned water molecule is activated, deacetylating the ß-lactam, while the ß-lactamase is regenerated and ready for the next catalytic cycle [8]. Thus, if the number of available carbapenemases is not limiting (this is not the case in our approach), the diagnostic performance should not be significantly influenced by the presence of two additional disks during the two-hour incubation step.

In their last paragraph, Muntean, A.-A. et al. correctly summarize the possible steps of a typical read-out process. As mentioned above, “carryover microsatellites”—grouped pin-point colonies of the read-out strain surrounding the carryover growth of the test strain—just identify a carbapenemase-positive test strain, even if a zone of inhibition above the cut-off appears, i.e., irrespective of the carbapenem type.

As indicated in the supplementary figure legend and in Section 4.3 of [5], this exemplifies our results, which were used in the definition of incubation conditions that ensure a detectable effect of *A. baumannii* group carbapenemases on ertapenem disks. Permeabilization was necessary to reach a sufficient concentration of carbapenemases in the trypticase soy broth, and an incubation time of 2 h gave the *A. baumannii* group carbapenemases sufficient time to exert their action [5].

The inclusion of positive and negative controls in every test round is stated in Paragraph 4.4 of the Methods section. The demonstration of expected results for the positive and negative controls indicates the integrity of the antibiotic disk used [5].

As always, the study results can only be expected to be reproduced if comparable conditions are employed, something clinical microbiologists are aware of, e.g., reagents from the same manufacturer applied in the same circumstances.
